# Supplementary material for: Efficacy of adding activity of daily living simulation training to traditional pulmonary rehabilitation on dyspnea and health-related quality-of-life
Source: PLoS One. 2020 Aug 27;15(8):e0237973. doi: 10.1371/journal.pone.0237973 (PMC7451521; doi:10.1371/journal.pone.0237973)
Supplement: S1 Table — (DOCX) [file pone.0237973.s001.docx]

S1 Table. Comparison of Patients who completed the Pulmonary Rehabilitation versus Drop-out

|  | **Completed, Energy Conservation PR**  **(n = 85)** | **Dropped-out, Energy Conservation PR**  **(n=53)** | **P-value, Completed vs. Dropped-out,**  **Energy Conservation PR** | **Completed,**  **Traditional PR**  **(n = 91)** | **Dropped-out,**  **Traditional PR**  **(n=51)** | **P-value, Completed vs. Dropped-out,**  **Traditional PR** |
| --- | --- | --- | --- | --- | --- | --- |
| **Age, years (**mean ± SD) | 70.0 ± 10.7 | 65.8 ± 9.83 | 0.02 | 66.9 ± 11.5 | 67.1 ± 10.0 | 0.94 |
| **Male (%)** | 50.6 | 39.6 | 0.21 | 47.1 | 48.4 | 0.88 |
| **COPD (%)** | 58.8 | 69.8 | 0.19 | 53.8 | 64.7 | 0.21 |
| Abbreviations: PR = Pulmonary Rehabilitation, COPD = Chronic Obstructive Pulmonary Disease | | | | | | |
