# Supplementary material for: Efficacy of adding activity of daily living simulation training to traditional pulmonary rehabilitation on dyspnea and health-related quality-of-life
Source: PLoS One. 2020 Aug 27;15(8):e0237973. doi: 10.1371/journal.pone.0237973 (PMC7451521; doi:10.1371/journal.pone.0237973)
Supplement: S2 Table — (DOCX) [file pone.0237973.s002.docx]

| n1= Energy Conservation/ n2= Traditional PR | **Chronic Obstructive Pulmonary Disease** | | | **Non-Chronic Obstructive Pulmonary Disease** | | |
| --- | --- | --- | --- | --- | --- | --- |
|  | **n1/n2** | **Energy Conservation PR**  **Mean Change (**± SD**)** | **Traditional PR**  **Mean Change (**± SD**)** | **n1/n2** | **Energy Conservation PR**  **Mean Change (**± SD**)** | **Traditional PR**  **Mean Change (**± SD**)** |
| **CAT*** | 50/49 | -3.76 ± 6.94 | -0.49 ± 7.99 | 34/41 | -3.71 ± 6.61 | -0.78 ± 6.67 |
| **CRQ Dyspnea** | 50/49 | 4.38 ± 5.83 | 3.71 ± 6.84 | 34/41 | 5.26 ± 6.78 | 2.46 ± 7.86 |
| **CRQ Fatigue** | 50/49 | 2.70 ± 4.61 | 2.00 ± 3.95 | 34/41 | 3.29 ± 4.41 | 2.73 ± 5.43 |
| **MMRC** | 50/49 | -0.62 ± 0.88 | -0.29 ± 1.26 | 35/41 | -0.57 ± 1.38 | -0.34 ± 1.15 |
| **6MWT** | 47/47 | 46.80 ± 70.37 | 73.52 ± 105.30 | 33/40 | 49.55 ± 59.96 | 39.23 ± 113.45 |
| *P values =0.03 and 0.06 for COPD and non-COPD respectively for comparison of change from baseline between Energy conservation vs Traditional PR using T Test.  **Abbreviations**: PR= Pulmonary Rehabilitation, CAT = COPD Assessment Tool, CRQ = Chronic Respiratory Questionnaire, MMRC = Modified Medical Research Counsel, 6MWT= Six Minute Walk Test | | | | | | |

S2 Table. **Comparison of the Change from Baseline in Patient Outcomes between Energy Conservation vs. Traditional Pulmonary Rehabilitation by COPD Status**
