# Supplementary material for: Efficacy of adding activity of daily living simulation training to traditional pulmonary rehabilitation on dyspnea and health-related quality-of-life
Source: PLoS One. 2020 Aug 27;15(8):e0237973. doi: 10.1371/journal.pone.0237973 (PMC7451521; doi:10.1371/journal.pone.0237973)
Supplement: Attachment — Submitted filename: rebuttal letter-Mahoney-wu.docx [file pone.0237973.s004.docx]

August 4, 2020

PONE-D-20-05739
Efficacy of Adding Activity of Daily Living Simulation Training to Traditional Pulmonary Rehabilitation on Dyspnea and Health-Related Quality-of-life
PLOS ONE

Dear Editor and Reviewers,

Thank you for the opportunity to allow us to improve the manuscript. A draft with track changes in the manuscript was also submitted along with a clean version. Please see below an item to item response to the reviewer’s comments in red.

Reviewer #1: The manuscript called “Efficacy of Adding Activity of Daily Living Simulation Training to Traditional Pulmonary Rehabilitation on Dyspnea and Health-Related Quality-of-life” improved after revision but remain some details that needs the authors attention.

1. Methods:
Pag 5-6 – “…Out of the 142 patients enrolled between October 2016 and August 2017 (pre-implementation period), 51 (35.9%) patients…, These patients…and personal reasons.” It is Results, not Methods. I suggest the authors transfer that paragraph to the beginning of results.

Paragraph has been transferred to the beginning of the results.

2. Suggestion to reduce the study limitations – The authors said “The questionnaires used to assess HRQOL,15 dyspnea and fatigue were not validated for non-COPD patients”. Looking the table 1 the authors has 59 COPD patients on EC-PR group and 54 on TPR group, the others chronic lung disease together adds 46 individuals in each group. The authors might do the separately analyzes of data COPD and not COPD. Perhaps the authors could do a separate analysis of COPD and non-COPD data. In my opinion, this will make the result stronger.

A subgroup analysis on the patient self-reported outcomes by COPD status was added as a supplemental table 2 and to results: “Subgroup analysis by COPD status was described in Supplemental Table 2. The change from baseline in CAT scores was significantly greater for the Energy Conservation versus Traditional PR in the COPD group (p=0.03) and trended towards significance in the non-COPD group (p=0.06). The change from baseline in the remaining outcomes (CRQ-dyspnea, CRQ-Fatigue, MMRC, six-minute-walk test) was similar between the Energy Conservation and the Traditional PR groups in both the COPD and non-COPD patients.” In the results section, the regression analysis does not show that the Patient outcomes significantly changed by COPD status. This was added. “Regression analyses using multiplicative interaction did not show any of the above results to significantly differ by COPD status (all P values >0.25)” (last paragraph, p. 11 and first paragraph, p. 12).

As result, we also deleted the sentence from limitations of “The questionnaires used to assess HRQOL, dyspnea and fatigue were not validated for non-COPD patients”.

We are grateful for the insightful comments that made this a higher quality manuscript.

Sincerely,

Kayla Mahoney, MS &  Wen-Chih Wu, MD
